# Treatment of Melasma With Q-Switched Laser in Combination With Tranexamic Acid

**DOI:** 10.1155/drp/1883760

**Published:** 2025-02-05

**Authors:** Zirui Liu

**Affiliations:** Department of Anesthesia, Xiangya School of Medicine, Central South University, Changsha, Hunan, China

## Abstract

Melasma, a pigmentary disorder that particularly affects Asian women, has been clinically proven to respond effectively to combination therapy of Q-switched lasers and tranexamic acid (TXA), especially with the advancements in laser aesthetics in recent years. However, treatment outcomes can be influenced by factors such as the wavelength and spot size of the Q-switched laser, the route of administration for TXA (including injectable, oral, or topical), as well as the dosage and duration of treatment. This article presents 13 different combination approaches from six randomized controlled trials, indicating that oral administration of TXA in combination with a 1064 nm Q-switched laser is currently the most widely used and effective treatment approach.

## 1. Introduction

Melasma is a prevalent acquired hyperpigmentation disorder characterized by symmetrical dark patches on sun-exposed areas, such as the face, also known as “chloasma” or “butterfly mask” [1]. It primarily presents as uniform or irregular brown or grayish–brown patches on the face, particularly on the cheeks, forehead, upper lip, and chin, as well as the neck, arms, and other sun-exposed skin regions.

The development of melasma is often linked to various factors, including genetic predisposition, sun exposure, hormonal fluctuations (such as during pregnancy, oral contraceptive usage, or hormone replacement therapy), and certain medication usage [2]. Visible light, especially high-energy visible light (HEVL) and long-wave UVA (UVA1), play a crucial role in the pathophysiology of melasma [3]. Women are especially prone to melasma, notably during pregnancy or menopause [4]. Melasma exhibits five primary pathomechanisms, namely, (1) abnormal activation of melanocytes, (2) deposition of melanin and melanosomes in the dermis and epidermis, (3a) increased mast cell presence and (3b) solar elastosis, (4) alterations in the basement membrane, and (5) heightened vascularization [5].

Melasma is typically a benign dermatological condition; however, it can induce psychological distress and emotional burden due to its impact on appearance [2]. As a result, many individuals seek treatment options to alleviate or diminish the appearance of the patches.

There are diverse treatment modalities available for melasma, such as the topical application of depigmenting agents, superficial chemical peels, laser treatments, radiofrequency therapy, and chemical peels, among others [6]. The optimal treatment approach varies based on individual circumstances and the severity of the condition. One commonly employed treatment method involves combining the use of tranexamic acid (TXA) with a Q-switched laser to achieve a reduction in melasma pigmentation.

In the 1950s, Japanese pharmacologist Utako Okamoto made a significant discovery when searching for a hemostatic drug: TXA, also known as transamin. TXA, a synthetic analog of lysine, serves as an antifibrinolytic agent. It competes with plasminogen activators to inhibit the activation of plasminogen and, at higher concentrations, noncompetitively blocks plasmin [7, 8]. As a result, TXA effectively prevents the dissolution and degradation of fibrin clots by plasmin [9]. Extensive research indicates that TXA can inhibit platelet activation induced by fibrinolysis during extracorporeal circulation, such as cardiopulmonary bypass (CPB) used in cardiac surgery [10]. Post-CPB bleeding involves multiple factors, and fibrinolysis is one of the few factors that can be mitigated through pharmacological intervention [11]. TXA also reduces excessive post-CPB bleeding through alternative mechanisms [12]. The binding affinity of TXA to plasminogen is 6–10 times stronger than that of ε-aminocaproic acid (ε-ACA) [13]. In animal models, TXA has demonstrated a dose-dependent increase in thrombus formation, contrary to its anticoagulant properties that inhibit thrombus formation [14, 15].

TXA is commonly used in the treatment of pigmentary skin diseases, notably melasma [16]. Its primary mode of action involves reducing the synthesis and deposition of melanin by inhibiting tyrosinase, a crucial enzyme in melanin synthesis. Tyrosinase facilitates the conversion of tyrosine to DOPA and DOPA to melanin [17]. Acting as a tyrosinase inhibitor, TXA disrupts tyrosinase activity and hampers the oxidation of dopaquinone, a critical step in melanin formation [14]. This interference results in a reduction in melanin synthesis, thereby alleviating or lightening pigmentary spots [18]. In addition, TXA exhibits anti-inflammatory effects, which can alleviate the inflammatory response triggered by pigmentation and improve the condition of pigmented skin by inhibiting the citric acid pathway [10, 19].

## 2. Mechanism and Application of Q-Switched Laser

A Q-switched laser is a laser device that produces high-energy, short-duration pulses. It employs a component known as a Q-switch to regulate the laser's output [20]. The Q-switch effectively accumulates energy within a brief timeframe and instantaneously releases it, resulting in the creation of a laser beam with high energy and a short pulse width. The amplification and output process of a Q-switched laser can be delineated into four pivotal stages: optical absorption, accumulation of excited states, Q-switch toggling, and laser emission. The crux of the Q-switched laser mechanism lies in the operational principle of the Q-switch [21]. By finely regulating the optical losses within the laser cavity, the Q-switch effectively governs the amplification and output of the laser. In its normal operational state, the Q-switch maintains substantial optical losses, impeding the amplification and release of the laser [22]. However, when laser output is necessitated, the Q-switch undergoes an abrupt state transition, reducing the optical losses and enabling the laser to rapidly amplify and emit an intense beam within an astonishingly brief duration [20].

Optical absorption: In the operation of a Q-switched laser, the active medium, such as an Nd:YAG crystal, undergoes optical absorption, wherein it assimilates external energy, predominantly in the form of light or electrical energy, and converts it into excited particles or electrons. Nd:YAG toning is a popular treatment protocol conducted on melasma patients and involves delivering low fluence, large spot size, and high-frequency pulses.

Excited state accumulation: Inside the laser cavity, particles or electrons in an excited state undergo persistent collisions and gradually amass energy, leading to a progressive increase in the population of the upper energy level [22].

Q-Switching: When the energy accumulation reaches a critical level, the Q-switch rapidly switches its state, reducing the optical losses within the laser cavity [20]. This enables the high-energy excited particles or electrons to rapidly decay to the ground state, initiating the stimulated emission process and triggering laser amplification and output.

Laser emission: Upon the switch-over of the Q-switch, the excited particles or electrons promptly return to the ground state, resulting in the emission of a laser beam with high energy and short pulse duration. This process occurs almost instantaneously, giving rise to the high-energy, short-pulsed output of a Q-switched laser [22].

After emitting high-energy, short-duration pulses of Q-switched laser beams, the pigmented deposits within the skin are subjected to bombardment, resulting in their fragmentation into smaller particles. This action induces changes in the surface properties of the pigment particles, promoting their uptake and breakdown by phagocytic cells, notably macrophages.

The 1064 nm Q-switched laser has strong penetration and low energy, which can reach melanocytes or black/blue dye particles in the deep skin. The 532 nm laser is more strongly absorbed by melanin, so the laser penetration of this wavelength is shallower, but the energy is higher, and the wavelength is strongly absorbed by hemoglobin. Due to the pathology of melasma primarily residing in the dermis or a mixed epidermal–dermal involvement, this treatment effectively targets facial pigmentation concerns. The low-fluence, Q-switched laser at 1064 nm, utilizing a multipass technique with a large spot size, is recommended as a treatment modality for melasma [23]. A meta-analysis conducted by the Guangzhou Institute of Dermatology, involving 358 patients, revealed no significant difference in the size of melasma and the severity index known as the melasma area and severity index (MASI) between the Q-switched laser monotherapy group and the medication group (*p*=57.1). In comparison to monotherapy with laser treatment, laser combined with medication showed a significantly greater improvement in MASI (*p* < 0.0001); however, no statistically significant results were found in terms of melanin index (MI) and self-assessment. When it comes to relative MASI (RMASI), the improvement in melasma was similar between the monotherapy with the laser group and the combination of lasers with other laser groups (*p*=56.3) [24]. Therefore, it indicates that the combination of Q-switched laser and TXA is currently the most effective treatment approach for melasma.

## 3. Efficacy of Q-Switched Laser Compared to and Combined With TXA in the Treatment of Ephelides and Melasma, Respectively

### 3.1. Intradermal Injection of TXA

A randomized controlled split-face study was conducted by Sayed, Tuqan, and Hilal from the Department of Dermatology at Cairo University. The study involved 30 female patients with bilateral facial melasma [25]. One side of the cheek received intradermal injections of TXA, while the other side underwent Q-switched laser treatment (532 nm). Changes in Pigmentation Area and Severity Score (PSI), MI, and erythema index (EI) were compared at baseline, 1 week posttreatment, and 2 months posttreatment. Statistical analysis indicated significant differences in the percentage changes of MI, PSI, and EI between the two treatments during and after the follow-up period (*p*=0.001). Immediately after treatment, PSI decreased by 66.5% and 15.4% on the laser and TXA sides, respectively. At the follow-up, PSI decreased by 69.4% and 26.1% on the laser and TXA sides, respectively. Q-switched laser treatment resulted in a 3.7% improvement in MI after treatment and 7.7% at follow-up, while TXA injections showed a 2.4% improvement after treatment and 6.5% at follow-up. It is worth noting that four patients who received Q-switched laser treatment experienced noticeable postinflammatory hyperpigmentation (PIH). In conclusion, the results suggest that Q-switched laser treatment alone is more effective than intradermal TXA injections alone for melasma treatment. It is worth noting that the author mainly studied Q-switched laser combined with TXA for the treatment of skin ephelides. Although the causes of freckles and melasma are different, freckles often have more genetic factors, but in view of the fact that ephelides and melasma are caused by glycated protein deposition, it is believed that ephelides and melasma are related. This paper focuses on the study of the treatment of Q-switched laser and TXA. Therefore, the Q-switched laser wavelength and parameters selected in the scope of this study are targeted at the removal of melasma.

A split-face comparative study was conducted by Hawwam, Ismail, and El-Attar from the Department of Dermatology and Venereology at Tanta University [26]. The study included 40 female patients with melasma. The right side of the face was treated with a combination of low-fluence Q-switched laser and intradermal TXA injections, while the left side received TXA injections alone. Clinical efficacy was assessed using the modified MASI (mMASI) score. The results revealed a significant reduction in mMASI scores on both sides of the face at 12 weeks and 24 weeks of treatment. At the end of the treatment, the mean ± SD decrease in mMASI score for the right side, which received combined intradermal TXA injections and Q-switched laser treatment, was 1.47 ± 1.53, which was statistically higher than the mean ± SD of 2.57 ± 1.50 for the left side treated with TXA injections alone. The combined treatment exhibited a statistically significant improvement in mMASI scores compared to TXA injections alone, indicating that the use of a Q-switched laser in combination with intradermal TXA injections is more effective.

Based on the two studies mentioned above, it can be concluded that the combination of Q-switched laser treatment and intradermal TXA injections is more effective in treating melasma compared to Q-switched laser treatment alone. In addition, Q-switched laser treatment alone is more effective than intradermal TXA injections alone in treating ephelides.

### 3.2. Oral TXA Therapy

A randomized controlled trial (RCT) conducted at the Faculty of Medicine, Ramathibodi Hospital, Mahidol University, enrolled 40 female patients with melasma [27]. The participants were divided into two groups: one group underwent Q-switched laser treatment using a 532 nm wavelength, and the other group received oral administration of 1500 mg of TXA daily. Evaluations including digital photography, skin microscopy, colorimetry, physician ratings, and patient satisfaction scores were performed at Weeks 2, 4, 6, and 12. Intriguingly, no statistically significant difference was observed, suggesting that the effectiveness of TXA alone and Q-switched laser treatment alone for melasma was comparable.

Another RCT conducted at the Faculty of Medicine, Alexandria University, involved 60 female patients with melasma [28]. The participants were divided into three groups: Group A received oral TXA 250 mg twice daily for 3 months, Group B received a combination treatment of topical TXA and 4% hydroquinone (HQ) cream, and Group C underwent a combination treatment of topical TXA and two sessions of low-fluence Q-switched 1064 nm laser with a 4-week interval. Monthly follow-ups were conducted for a total of 9 months. Following the discontinuation of treatment, Group B exhibited the lowest average mMASI score (2.34 ± 2.37), followed by Group A (6.38 ± 4.04) and Group C (7.24 ± 4.95).

However, there is also literature indicating that HQ is less effective than TXA in treating melasma and is more likely to lead to drug tolerance [29]. Generally speaking, topical TXA is considered less effective compared to HQ or oral TXA. Therefore, based on perceived treatment outcomes, doctors may prefer oral TXA [6].

### 3.3. Comparison of Oral and Intradermal Injection of TXA and Other Treatments

A randomized study conducted by Behrangi et al. from the School of Medicine, Iran University of Medical Sciences, included a sample size of 40 patients, with 20 patients in each treatment group [30]. Six laser treatments were performed every 2 weeks. In one group, 20 patients received local microinjections (4 mg/mL) of TXA and Q-switched 1064 nm laser, while in the other group, 20 patients received oral TXA 250 mg three times daily and Q-switched 1064 nm laser every 2 weeks. Both groups showed significant reductions in MASI scores and ΔE during the study period (*p* < 0.001). Interestingly, there were no significant differences in MASI scores (*p*=0.99) and ΔE (*p*=0.53) between the two groups over time, with no statistically significant differences observed (*p* > 0.05).

A study conducted by Su Jung Park and colleagues from the Department of Dermatology, Chung-Ang University College of Medicine, included 25 patients with melasma [31]. One side of the face was treated with low-energy Q-switched 1064 nm laser alone, while the other side of the face was treated with a combination of laser treatment and a topical mixture of TXA, niacinamide, and quercetin. Overall improvements were evaluated using the mMASI score. Although both sides of the face showed clinical improvement, the combination treatment demonstrated greater improvement in terms of the average half MASI score compared to laser treatment alone. The study suggests that a combination of a Q-switched 1064 nm laser and a topical mixture of TXA, niacinamide, and quercetin is a good choice for treating melasma.

Extensive clinical data suggest that there is no apparent disparity in clinical effectiveness between administering TXA orally or via intradermal injection [32]. Taking into account its noninvasive nature and greater convenience for patients, oral TXA has emerged as the most efficient option, particularly in cases of stubborn melasma. However, it has been known to cause gastrointestinal discomfort and menstrual irregularities in many individuals [33]. Prior to prescribing this medication, it is crucial to consider its prothrombotic properties and other potential side effects. Intralesional injection and localized TXA microneedling have proven to be viable alternatives to oral administration, and combining TXA with other cosmeceuticals can enhance treatment outcomes [29]. In addition, topical TXA has demonstrated superior tolerability compared to HQ, a conventional topical therapy for melasma. The sources, research methods, and main results of the six studies mentioned above are presented in tabular form in Table 1.

## 4. Discussion

### 4.1. Color Deposition and Recurrence

When treating pigmentary skin conditions such as melasma or pigmented spots, a Q-switched laser may potentially induce a phenomenon known as PIH. The action of laser energy can cause a certain degree of damage and inflammatory response to the skin while targeting the pigment particles. Following laser treatment, during the healing process, the body's inflammatory response releases various signaling molecules and cytokines that may promote the activation of melanocytes (skin pigment cells) and an increase in melanin production. This results in pigment deposition and exacerbation of spots. Another key mechanism that causes pigmentation is the release of melanin, usually due to the destruction of keratinocytes. This melanin is then captured by the melanophage, which is usually abundant in the dermis and does not depend solely on the epidermal melanin. This is one of the reasons why PIH can be so persistent in some individuals [34].

PIH following Q-switched laser treatment is typically a temporary phenomenon, which tends to gradually lessen or fade over time. However, for certain individuals, especially those with more sensitive skin, it may take longer to restore normal pigment uniformity. Similarly, Q-switching laser treatment may also cause leopard-like vitiligo symptoms due to cytochrome loss, which can only be treated by ultraviolet irradiation, although relatively rare, but it also reminds doctors and patients that before choosing laser treatment, they should choose the appropriate wavelength and course of treatment, so as to reduce the occurrence of adverse complications [6]. This interference with the correct assessment of the effectiveness of Q-switched laser in treating melasma poses challenges for both doctors and patients. It is also important to note that recurrence after discontinuing laser treatment is very common [23]. Patients often have the ultimate goal of a complete cure, unaware that melasma can only be managed or mitigated but not completely eradicated.

However, PIH can be prevented by routinely performing a spot test and selecting the appropriate therapeutic parameters based on the endpoint response (subpurpura dose is recommended). Those with dark skin choose low energy, long pulse duration, and long wavelength (1064 nm). Intraoperative and postoperative cooling: Postoperative routine cold compress, if the postoperative reaction is serious, can be alleviated by the cold compress method, at the same time to be vigilant about the occurrence of frostbite. Moisturize well after surgery to avoid scab. If scabs form, they should be delayed through strict sun protection, mainly by applying sunscreen, wearing hats, umbrellas, wearing sunscreen masks, and other hard sun protection methods. If there are burns and blisters, refer to the vascular laser postoperative care method to create a wet healing environment. The blisters are large and can be broken and drained with a syringe needle. Pay attention to preserve the blister wall, and if necessary, apply an antibiotic ointment to prevent infection. Topical application of steroids after surgery can alleviate the occurrence of PIH.  Table 2 shows the prevention methods of PIH mentioned in the paper.

At the same time, it is worth noting that some patients use NdYAG toning for a long time in pursuit of curative effects, which may aggravate pigmentation and cause unsatisfactory therapeutic effects [6]. They frequently discontinue treatment upon observing improvement and are unwilling to continue with maintenance therapy. This is also a factor that requires doctors to explain to patients and obtain their understanding. The topical lightening agents most frequently prescribed and extensively researched for melasma include HQ, azelaic acid (AzA), and retinoids. However, it is important to note that only HQ and a specific triple combination cream (Tri-Luma; containing fluocinolone acetonide 0.01%, HQ 4%, and tretinoin 0.05%) have received approval from the US Food and Drug Administration (FDA) [35]. Consequently, patients have very limited options in treating pigment deposition, and in some economically underdeveloped regions, there may be varying quality of generic drugs that can interfere with patients' treatment.

### 4.2. The Use of TXA May Cause Some Side Effects

TXA, as an antifibrinolytic medication, is primarily used to treat coagulation disorders. Therefore, when using TXA to treat melasma, it may cause certain side effects in patients [11]. (1) Gastrointestinal discomfort: This includes nausea, vomiting, gastric burning or discomfort, and diarrhea, which are related to the digestive system [36]. (2) Skin allergic reactions: In rare cases, the use of TXA may cause skin allergic reactions such as rashes, hives, and itching [37]. (3) Thrombotic events: Although TXA is used to control bleeding, in extremely rare cases, it may increase the risk of thrombosis. This can lead to vascular-related issues such as venous thrombosis, deep vein thrombosis, or pulmonary embolism [38]. (4) Menstrual changes: In women, the use of TXA may result in amenorrhea, making it important to exercise caution when prescribing the medication to patients who are planning to conceive or have a desire to become pregnant [36]. (5) Cognitive impairment: In some individuals, TXA may cause symptoms of fatigue, dizziness, or difficulty concentrating [38]. (6) Muscle or joint pain: This is a relatively rare side effect that may manifest as muscle or joint pain [38]. The possible side effects of TXA are shown in Table 3.

Considering the potential side effects of TXA, it is typically prescribed in clinical treatment for a period of 2 months, followed by a 1-month break, and then another 2-month course of treatment [7].

### 4.3. Prospects for New Drug Combinations

Many topical cream medications have been proven to be effective in treating melasma. For example, a triple combination cream, consisting of a moderate-potency corticosteroid (fluocinolone acetonide 0.01%), a retinoid (tretinoin 0.05%), and HQ 4%, is one of the widely used topical treatments for melasma worldwide. Furthermore, research by Abdelaal M. Elkamshoushi suggests that the combination of 4% HQ with TXA may even have better efficacy than the combination of Q-switched laser with TXA [28]. Topical TXA can be combined with skin-lightening agents and antioxidants, such as niacinamide, which itself possesses whitening properties [39, 40]. It can also be used in conjunction with lactobionic acid to enhance skin cell renewal [41]. In addition, certain herbal products have shown anti-inflammatory and skin-lightening effects, although their specific actions require further evaluation [6]. Herbal remedies have also demonstrated promising therapeutic effects [42].

Melasma has a large patient population, particularly in Asian and East Asian countries. In the past, patients with melasma often neglected or believed it was untreatable [6]. With the continuous development of laser treatment technology and people's pursuit of beauty, it is believed that there will be more literature available for researchers in the future, aiming to discover the optimal diagnostic and therapeutic approaches that minimize damage and provide more long-lasting efficacy for patients.

## 5. Conclusion

Undoubtedly, the use of Q-switched lasers and TXA for the treatment of melasma has been clinically proven to be effective. However, factors such as the wavelength and spot size of the Q-switched laser, the route of administration for TXA (including injectable, oral, or topical), as well as the dosage and duration of treatment, can impact treatment outcomes. This article presents 13 different combinations from six RCTs, suggesting that oral TXA combined with a 1064 nm Q-switched laser is currently the most widely used and effective treatment approach.

Furthermore, there is still a shortage of RCTs evaluating the combination of Q-switched lasers and TXA for melasma treatment, and the available data for meta-analysis are quite limited. Therefore, it requires collaborative efforts from researchers worldwide to identify the optimal diagnosis and treatment approach for melasma based on clinical data.

## Figures and Tables

**Table 1:** A summary table of the methods used and the results evaluated for the two studies mentioned above.

Research source	Research method	Evaluation
Khadiga S. Sayed	A: Intradermal injections of TXA B: Q-switched laser treatment (532 nm)	B is better
Soha Abdalla Hawwam	A: Low-fluence Q-switched laser and intradermal tranexamic acid (TXA) injections B: TXA injections alone	A is better
Rutnin S (Mahidol University)	A: Q-switched laser treatment using a 532 nm wavelength B: Oral administration of 1500 mg of TXA daily	No significant difference
Elkamshoushi AM (Alexandria University)	A: Oral TXA 250 mg twice daily for 3 months B: Topical TXA and 4% hydroquinone cream C: Topical TXA and two sessions of low-fluence Q-switched 1064 nm laser with a 4-week interval	C is the best and A is better than B
Elham Behrangi	A: Local microinjections (4 mg/mL) of tranexamic acid and Q-switched 1064 nm laser. B: Oral tranexamic acid 250 mg three times daily and Q-switched 1064 nm laser every 2 weeks.	No significant difference.
Su Jung Park	A: Low-energy Q-switched 1064 nm laser alone B: Laser treatment and a topical mixture of tranexamic acid, niacinamide, and quercetin.	B is better.

**Table 2:** Summary table of prevention methods for PIH.

Prevention method of PIH
1. Routinely performing a spot test and selecting the appropriate therapeutic parameters
2. Those with dark skin choose low energy, long pulse duration, and long wavelength (1064 nm)
3. Intraoperative and postoperative cooling: Postoperative routine cold compress, if the postoperative reaction is serious, can be alleviated by the cold compress method, at the same time to be vigilant about the occurrence of frostbite
4. Moisturize well after surgery to avoid scab. If scabs form, they should be delayed through strict sun protection methods, mainly by applying sunscreen, wearing hats, umbrellas, wearing sunscreen masks, and other hard sun protection methods
5. If there are burns and blisters, refer to the vascular laser postoperative care method to create a wet healing environment
6. The blisters are large and can be broken and drained with a syringe needle
7. Pay attention to preserve the blister wall, and if necessary, apply an antibiotic ointment to prevent infection
8. Topical application of steroids after surgery can alleviate the occurrence of PIH

**Table 3:** Summary of TXA side effects.

Side effect	Detail
Gastrointestinal discomfort	Nausea, vomiting, gastric burning or discomfort, and diarrhea
Skin allergic reactions	Skin allergic reactions
Thrombotic events	Venous thrombosis, deep vein thrombosis, or pulmonary embolism
Menstrual changes	Amenorrhea risks
Cognitive impairment	Symptoms of fatigue, dizziness, or difficulty concentrating
Muscle or joint pain	Relatively rare may manifest as muscle or joint pain

## Data Availability

The data that support the findings of this study are available from the corresponding author upon reasonable request.
